# The Role of NBI HDTV Magnifying Endoscopy in the Prehistologic Diagnosis of Laryngeal Papillomatosis and Spinocellular Cancer

**DOI:** 10.1155/2014/285486

**Published:** 2014-06-17

**Authors:** Petr Lukes, Michal Zabrodsky, Eva Lukesova, Martin Chovanec, Jaromir Astl, Jaroslav A. Betka, Jan Plzak

**Affiliations:** Department of Otorhinolaryngology, Head and Neck Surgery, First Faculty of Medicine, Charles University in Prague and University Hospital Motol, V Úvalu 84, 150 06 Prague 5, Czech Republic

## Abstract

Narrow band imaging (NBI) HDTV (high definition television) magnifying endoscopy is considered to be superior for the accurate display of the microvascular patterns of superficial mucosal lesions. Observation of changes in intraepithelial papillary capillary loops (IPCL) can help distinguish benign from malignant lesions as part of an “optical biopsy.” However, IPCL changes in papillomas may be mistaken for spinocellular cancer (SCC). The aim of the study was to determine whether observing microvascular changes alone is sufficient for discriminating between laryngeal SCC and papillomatosis. An additional aim was to identify associated characteristics that could clarify the diagnosis. The study included 109 patients with a suspected laryngeal tumor or papilloma. HDTV NBI magnifying endoscopy was performed during direct laryngoscopy. It was possible to visualize IPCL changes in 82 out of 109 patients (75.2%). In 71 (86.6%) patients, the diagnosis was correctly determined. In 4 (4.9%) cases, the diagnosis of SCC was expressed on the basis of finding pathologic IPCL, but histology did not demonstrate malignancy. To achieve a correct diagnosis using HDTV NBI magnifying endoscopy, it is important not only to observe changes in the shape of IPCL but also to note possible papillary structures with central-axis capillaries typical of papillomatosis.

## 1. Introduction

Narrow band imaging (NBI) is an optical image enhancement technology that enhances vessels in the surface of mucosa using the characteristics of the light spectrum [1]. The NBI system consists of the same components as conventional videoendoscopic systems: a light source, a camera unit, and a camera head or chip-equipped videoendoscope. Additionally, the NBI system contains a special image processor and a lighting unit with special filters that narrow the frequency range of emitted light to 400–430 nm (centered at 415 nm) and 525–555 nm (centered at 540 nm) bands. The emitted light has less penetration and less scattering and is highly absorbed in hemoglobin, thus enhancing the image resolution. The reflection is captured by a charge-coupled device chip (CCD), and an image processor creates a composite pseudocolor image, which is displayed on a monitor, enabling NBI to enhance mucosal contrast without the use of dyes [2, 3].

The detection of surface mucosal changes that are characteristic of neoplastic lesions (e.g., dysplasia, in situ carcinoma, and carcinoma), epithelial abnormalities (thickening and changes in the surface layer), and vascular changes can be best achieved with NBI. Changes in intraepithelial papillary capillary loops (IPCL), such as dilatation, weaving, and alterations in caliber and shape, occur in developing neoangiogenesis. These changes are visible as typical brown dots.

In the larynx, conventional (nonmagnifying) NBI endoscopy reaches a sensitivity of 61–91% and a specificity of 87–92% [4, 5]. The most frequent false positive results were reported in cases of laryngeal papillomatosis, where the microvascular pattern could be misinterpreted as malignant neoplasia [5].

Combination of NBI with HDTV (high definition television) and magnifying endoscopy is being used recently. Zooming flexible videoendoscopes are available for gastroenterology. Endoscopic systems can achieve ultrahigh (up to 150-fold) magnification. This magnification makes it possible to visualize the microstructure of the IPCL pattern [6, 7]. This approach is promising for better discrimination of malignant and benign lesions as part of “prehistologic diagnosis” or “optical biopsy” [8]. Nevertheless, there is no ultrathin, zooming, flexible videoendoscope available for ENT purposes; therefore, a combination of rigid telescopes and an HDTV camera head must be used to achieve sufficient resolution and ultrahigh magnification [9].

There are several papers dealing with the correlation of IPCL changes and the extent of cancer proliferation. They state that the depth of tumor invasion can be determined according to the degree of IPCL changes [7]. The first classifications of IPCL changes were created for the esophagus [6] and the oral cavity [10].

A new classification of the stages of IPCL changes was proposed for laryngeal superficial mucosal lesions [11]. The authors suggest that the in vivo differentiation of nonmalignant from malignant laryngeal lesions can be performed by evaluating the morphology of mucosal capillaries.

The classification is based solely on IPCL shape changes. Neoangiogenesis followed by corresponding IPCL changes may occur not only in cancers but also in inflammation, wound healing, and so on [12]. NBI was proven to be a useful method for the diagnosis of recurrent respiratory papillomatosis [13]. Nevertheless, IPCL changes that are observable in papillomatosis can often mimic changes in the tumor and thus their recognition may be difficult [9]. The presence of multiple papillae covered by squamous epithelium with a vessel along a central axis of each papilla is typical for laryngeal papillomatosis [14]. The question is whether the observation of IPCL changes as the only marker can be sufficient for achieving a “prehistologic diagnosis” in diagnostics of laryngeal lesions.

As the NBI endoscopy approach is based on the observation of the mucosal surface, situations where it is impossible to see the clear mucosal surface (e.g., ulceration, hyperkeratosis, blood, etc.) can prevent the valid evaluation of the findings [9].

The aim of this study was to determine the effectiveness of HDTV NBI magnifying endoscopy for the intraoperative differentiation between laryngeal carcinoma and laryngeal papillomatosis. An additional aim was to identify the associated endoscopic characteristics that could help clarify the diagnosis.

## 2. Materials and Methods

The present study was conducted between May 2010 and July 2013 at the Department of Otorhinolaryngology, Head and Neck Surgery, First Faculty of Medicine, Charles University in Prague, University Hospital Motol, Prague, Czech Republic. A total of 109 patients (95 males, 14 females, age of 21–83 years, and mean age of 61 years) were enrolled. All these patients were sent to the department with findings of a suspected laryngeal tumor or a suspected laryngeal papilloma. A biopsy was not previously taken in any of the patients. Patients with clearly benign lesions (nodules, polyps, edemas, or granulomas) were excluded. The study was approved by an authorized medical ethics committee, and informed consent was obtained from each patient prior to inclusion. All patients underwent direct laryngoscopy under general anesthesia. The NBI HDTV magnifying endoscopy was performed in all of the patients during the direct laryngoscopy.

### 2.1. NBI HDTV Magnifying Endoscopy

**Figure 1:**
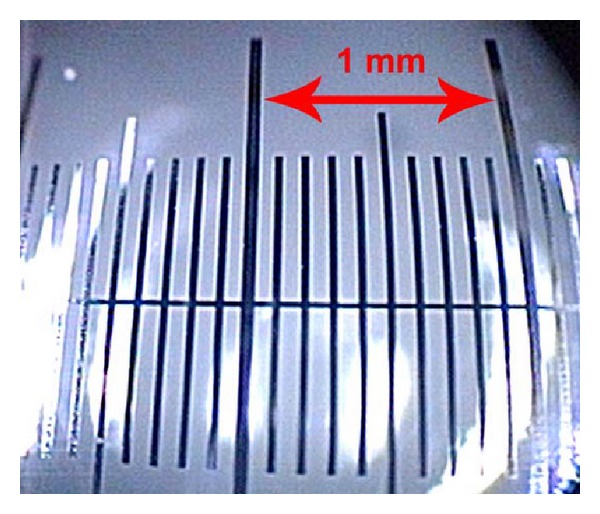
Calibrated scale viewed by the endoscopic magnification system; the smallest segment represents 0.1 mm.

The OLYMPUS EXERA II HDTV system was used in combination with the OLYMPUS OTV-S7ProH-HD-12E camera head and OLYMPUS rigid telescopes of 0°, 30°, and 70° (Olympus Medical Systems, Tokyo, Japan). To achieve the maximum magnification and resolution, the focus of the camera was set to maximum close-up, and the visualization was performed from a distance of less than 1 mm from the mucosal surface. When using the monitor OLYMPUS OEV-191H (Olympus Medical Systems, Tokyo, Japan), a 150-fold magnification can be achieved. This magnification was confirmed using a calibrated scale OBM-Stereo 40/400 0.1 mm (OLYMPUS CZECH GROUP, Prague, Czech Republic); see Figure 1.

### 2.2. Evaluation of Findings

**Figure 2:**
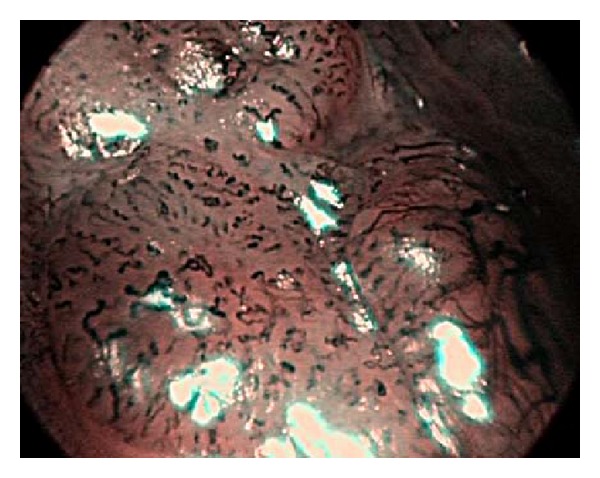
Features typical of a carcinoma: dilatation, weaving, changes in caliber, and variety of IPCL shapes, are visible.

**Figure 3:**
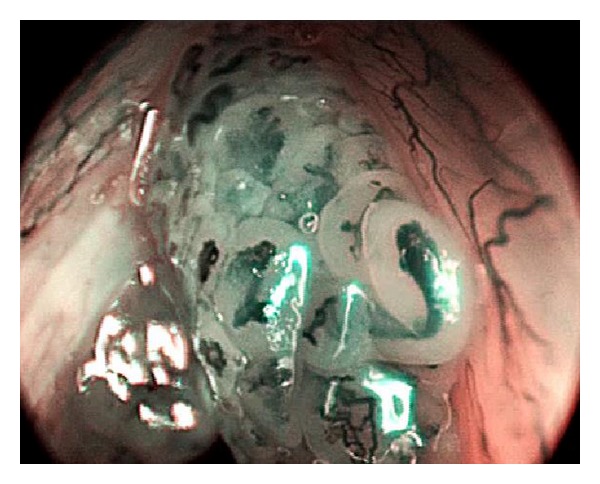
Features typical of a papilloma: irregular shapes, calibers, and IPCL courses with regular papillae with central vessels, are visible.

The lesions were observed using a NBI mode. The primary goal was to observe the IPCL under maximum magnification. In the cases where IPCL were observable, the observations of irregular shapes, calibers, and courses of the IPCL were assessed for potential carcinomas (Figure 2). In cases where similar changes were observed in addition to the structures of papillae with a central capillary loop, the lesions were diagnosed to be papillomas (Figure 3).

**Figure 4:**
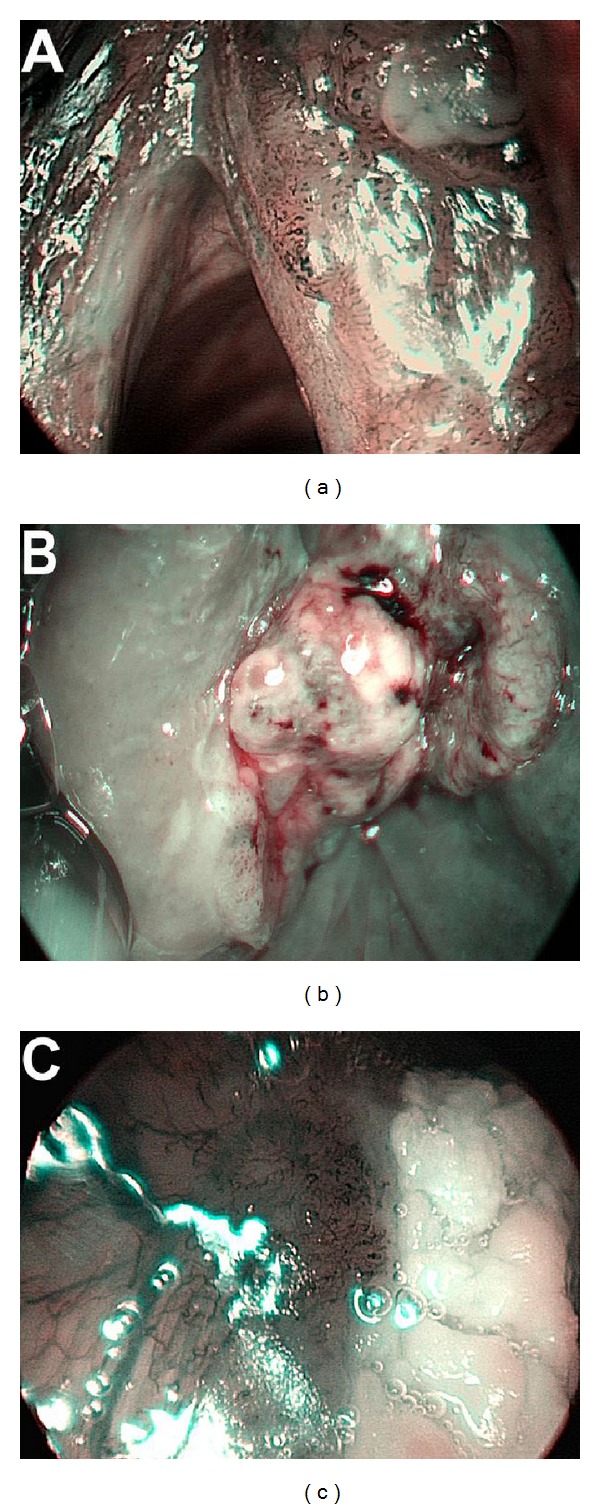
Surface of the lesion: (a) smooth, (b) rough, and (c) hyperkeratotic.

**Figure 5:**
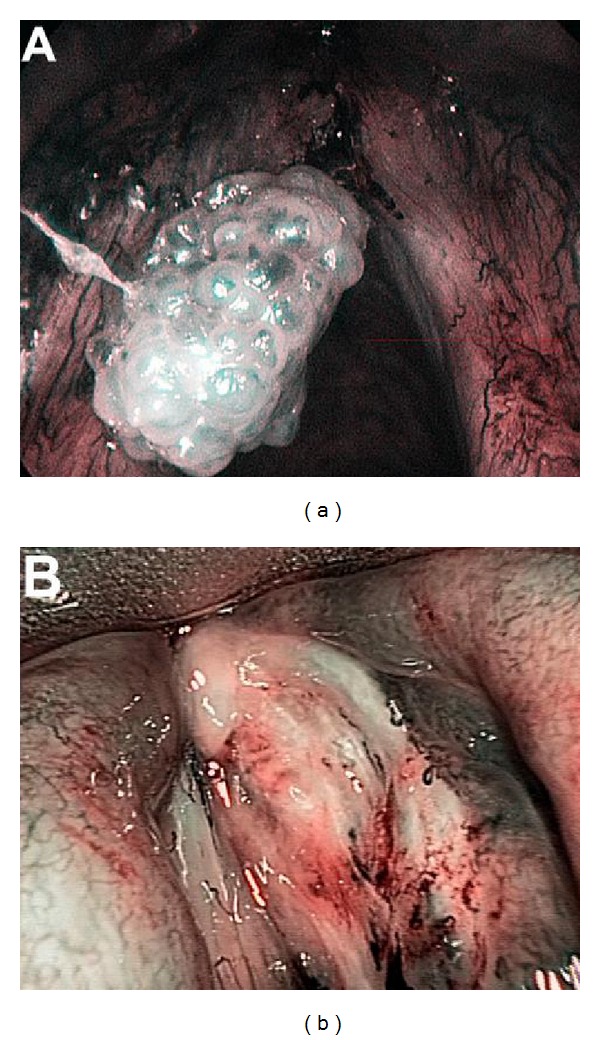
Behavior of the lesion: (a) exophytic lesion and (b) ulcerative lesion.

**Figure 6:**
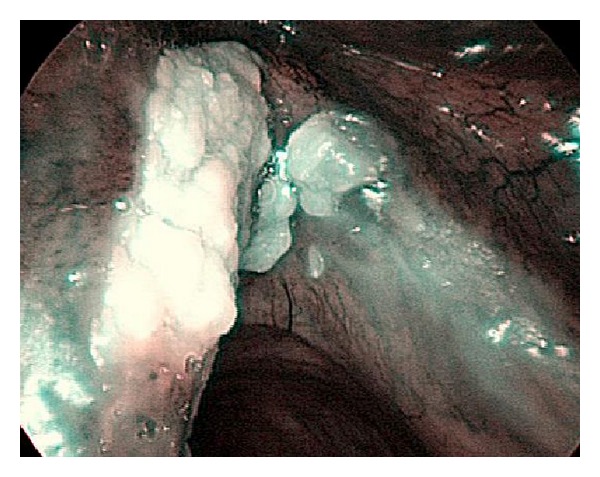
Lesion extending to both vocal folds.

**Figure 7:**
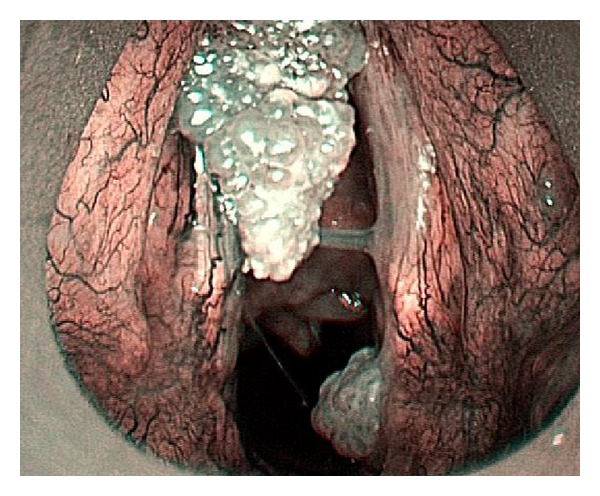
Multiple lesions.

**Figure 8:**
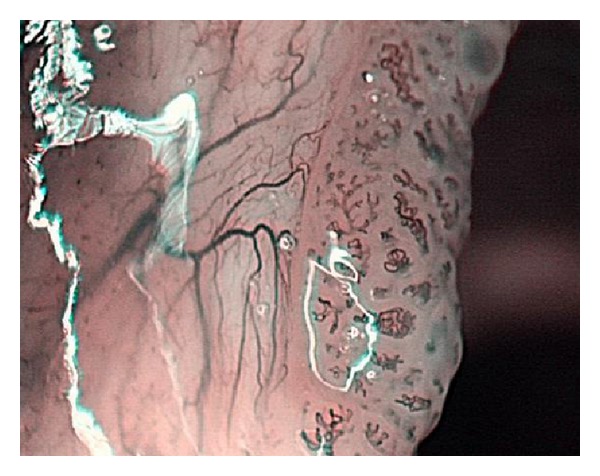
A branching feeding vessel.

To determine other signs that could further improve the diagnosis, the characteristics of the lesions were described as follows:

- surface of the lesion (smooth/rough/hyperkeratosis) (Figure 4),
- behavior (exophytic or ulcerative) (Figure 5),
- laterality (unilateral/bilateral) (Figure 6),
- multiplicity (single/multiple lesions) (Figure 7),
- branching feeding vessel (present/absent) (Figure 8).

In the cases of bleeding from the lesion, a cotton swab soaked in adrenaline was used to stop the bleeding and to cleanse the mucosa. In the cases of a thick layer of hyperkeratosis or fibrin covering the vascular pattern, the IPCL were observed at the edge of the lesion. Images and video sequences were recorded for further analysis. A biopsy of each lesion was performed. Tissue specimens were fixed in 10% formalin for histopathological analysis.

### 2.3. Statistical Analysis

Data analysis was performed with the R Project for Statistical Computing software environment, version 2.15.2 (http://www.r-project.org/).

## 3. **Results**

It was possible to clearly observe IPCL in 82 out of 109 patients (75.2%). In 27 patients (24.8%), it was impossible to observe IPCL structure due to hyperkeratosis, ulceration, bleeding, or fibrin. The diagnosis was correctly determined in 71 (86.6%) out of 82 patients with observable IPCL, according to the differentiation of IPCL changes and observations of papillary arrangement of the lesions. Of those, 39 were SCCs and 32 were papillomas. These results were confirmed using histology.

In 7 patients (8.5%), it was not possible to determine whether the findings supported a diagnosis of SCC or papillomatosis using observations of IPCL and papillary arrangement. In these 7 cases, histology showed that 4 were SCCs, 2 were papillomas, and 1 was a case of dysplasia. In 4 patients (4.9%), the diagnosis was a false positive. The lesions were identified as SCCs according to IPCL changes; however, histologically, 1 case was shown to be dysplasia, and the other 3 were benign lesions with no signs of malignant changes.

In the cases when IPCL were visible, the sensitivity and specificity for SCC were 100% and 82%, respectively, and for papillomatosis sensitivity and specificity were 94% and 100%, respectively.

Of 27 lesions with IPCL not observable, histology results revealed that 11 were SCCs, 10 were dysplasia, and 6 were benign lesions.

The results of the associated endoscopic characteristics analysis are displayed in Tables 1, 2, 3, and 4. In the group where the diagnosis was set by IPCL changes, statistically significant associated endoscopic characteristics were as follows:

1. surface of the lesion (smooth/rough/hyperkeratosis) (Fisher's Exact Test, *P* = 0.000000003641);
2. laterality (Fisher's Exact Test, *P* = 0.0000002364);
3. multiplicity (Fisher's Exact Test, *P* = 0.0000005575).

In the group where the diagnosis was not possible using changes in IPCL and in the group of false positive results, no significant differences were found when evaluating associated endoscopic characteristics.

In the group where IPCL changes were not visible, a statistically significant associated endoscopic characteristic was the presence of a feeding vessel (Fisher's Exact Test, *P* = 0.0004264).

Statistical analysis of the frequency of associated endoscopic characteristics in the whole study cohort, without regard to the IPCL, yielded the following results:

- surface of the lesion (smooth/rough/hyperkeratosis); hyperkeratosis and rough surfaces were observed most frequently in SCCs, which was shown to be statistically significant (Fisher's Exact Test, *P* = 0.00000000000357),
- behavior (exophytic or ulcerative); no statistically significant differences were found (Fisher's Exact Test, *P* = 0.071),
- laterality (unilateral/bilateral); bilateral lesions were most frequently observed in papillomatosis, which was statistically significant (Fisher's Exact Test, *P* = 0.0000107),
- multiplicity (single/multiple lesion); multiple lesions were observed most frequently in papillomatosis, which was statistically significant (Fisher's Exact Test, *P* = 0.000000726),
- branching feeding vessel (present/absent); feeding vessels were most frequently observed in benign lesions, which was statistically significant (Fisher's Exact Test, *P* = 0.0171).

## 4. Discussion

NBI is an endoscopic method with a high degree of sensitivity and specificity. Clinical discrimination of papillomatosis and early stage of laryngeal SCC can be difficult using conventional (nonmagnifying) NBI endoscopy. Recently, NBI endoscopy has been increasingly used in combination with magnification and high resolution systems (HDTV). It is known that the growth of epithelial tumors leads to escalated neoangiogenesis [12]. In practice, this phenomenon can be observed as changes in the IPCL arrangement, diameter, and shape and as a loss of regularity [6].

To visualize these changes successfully, the endoscopic system must achieve not only a sufficient resolution but also the required magnification. The method described in this paper allows one to achieve up to 150-fold magnification intraoperatively and can therefore be described as magnifying endoscopy. The biological behavior of the lesion can be identified using the nature of IPCL changes. IPCL changes that are observable in laryngeal SCC may be, however, very similar to the changes that are apparent in recurrent respiratory papillomatosis, where there is also a potential for increased neoangiogenesis [15].

A new classification of the stages of IPCL changes was proposed for laryngeal superficial mucosal lesions [11]. This classification was only based on IPCL shape changes and seems to be insufficient to achieve the best results possible or to improve the potential of endoscopic “prehistologic diagnosis.”

The usefulness of NBI in the detection of recurrent respiratory papillomatosis was described by Tjon Pian Gi et al. [13]. According to our results, the observation of not only the changes in the behavior of IPCL but also the structure of the epithelium surface, where, in most of the cases of papillomatosis, regular multiple papillae with vessels along the central axis of each papilla can be found, is the most important feature for distinguishing between papillomas and SCCs. On the other hand, the vascular pattern of SCC shows many irregularities and a partial or complete collapse of vascular microarchitecture can be frequently observed.

When all of these characteristics are taken into account, SCCs can be accurately distinguished from papillomas and the diagnosis can be made before histology results are available. Further development of HDTV NBI magnifying endoscopy is needed so that a real “prehistologic diagnosis” or “optical biopsy” can be achieved. It should be taken into consideration, however, that because NBI is a method based on the observation of vascular changes in the mucosa, in cases where the surface of the lesion prevents a direct view of the mucosa (ulcer, hyperkeratosis, etc.), this method is limited [9].

In this study, it was not possible to observe the IPCL in the lesions of 24.8% of the cases. In 75.2% of the cases, it was possible to accurately display the surface of the lesion, and 86.6% of the cases were diagnosed correctly before the histology results were obtained. Sensitivity and specificity for SCC were 100% and 82%, respectively, and for papillomatosis sensitivity and specificity were 94% and 100%, respectively. In papillomas, IPCL changes were similar to those visible in cancers. This finding is in accordance with data published by Bolontrade et al. [16], who found that an increase in the density and size of blood vessels can be observed in both late papillomas and carcinomas.

In this study, the observations of papilliform structures of the epithelium combined with observations of IPCL were shown to be crucial for setting the diagnosis. For the discrimination of laryngeal SCC from recurrent respiratory papillomatosis, both the changes in IPCL and the changes of the epithelium surface must be taken into account. For papillomatosis, the typical papillary arrangement of the epithelium with a central-axis capillary in each papilla is usually present. The shape of the capillaries, however, may undergo changes similar to those observed in cancer. This study was also focused on finding other associated, endoscopically observable characteristics that can improve the accuracy of endoscopic diagnosis.

Features significant for the improvement of endoscopic diagnosis were observation of surface of the lesions (in papillomas, a smooth surface was found in all cases, whereas carcinomas may show hyperkeratosis or a rough surface), laterality, and multiplicity of the lesions. Additionally, lesions affecting both vocal cords and multiple lesions were most frequently observed in papillomatosis.

Surprisingly, the presence of branching feeding blood vessels was most often visible in benign diagnoses. Nevertheless, this result may be influenced by very low numbers of benign lesions in the study group.

## 5. Conclusions

HDTV NBI magnifying endoscopy is a method with a high accuracy for the diagnosis of laryngeal lesions that can be available before the histology results. The most important observation for a correct diagnosis is that of the changes in the shape of IPCL. For distinguishing between SCC and papillomatosis, it is crucial to also focus on the epithelial surface and to observe the papillary structures with a central-axis capillary and a more or less regular arrangement. Some of the associated endoscopic characteristics, such as the surface, the presence of multiple lesions, and the spread to both vocal cords, may be taken as the criteria used for a more accurate diagnosis.

## Figures and Tables

**Table 1:** Findings with the diagnoses performed by IPCL, associated endoscopic characteristics.

	SCC	Papillomatosis
Total	39	32

Exophytic lesion	34	32
Ulcerative lesion	5	0

Smooth surface	14	32
Rough surface	9	0
Hyperkeratosis	16	0

Feeding vessel present	8	10
Feeding vessel absent	31	22

Solitary lesion	39	15
Multiple lesion	0	17

Unilateral lesion	36	11
Bilateral lesion	3	21

**Table 2:** Findings with an unclear diagnosis using IPCL, associated endoscopic characteristics.

	SCC	Papillomatosis	Dysplasia
Total	4	2	1

Exophytic lesion	4	2	1
Ulcerative lesion	0	0	0

Smooth surface	3	2	1
Rough surface	1	0	0
Hyperkeratosis	0	0	0

Feeding vessel present	1	0	1
Feeding vessel absent	3	2	0

Solitary lesion	4	1	1
Multiple lesion	0	1	0

Unilateral lesion	2	1	0
Bilateral lesion	2	1	1

**Table 3:** False positive findings using IPCL, associated endoscopic characteristics.

	Dysplasia	Benign
Total	1	3

Exophytic lesion	1	3
Ulcerative lesion	0	0

Smooth surface	0	1
Rough surface	0	0
Hyperkeratosis	1	2

Feeding vessel present	1	0
Feeding vessel absent	0	3

Solitary lesion	1	1
Multiple lesion	0	2

Unilateral lesion	0	2
Bilateral lesion	1	1

**Table 4:** Findings with nonvisible IPCL, associated endoscopic characteristics.

	SCC	Dysplasia	Benign
Total	11	10	6

Exophytic lesion	9	10	5
Ulcerative lesion	2	0	1

Smooth surface	0	2	1
Rough surface	4	0	1
Hyperkeratosis	7	8	4

Feeding vessel present	1	3	6
Feeding vessel absent	10	7	0

Solitary lesion	9	7	6
Multiple lesion	2	3	0

Unilateral lesion	7	6	5
Bilateral lesion	3	4	1

## References

[1] Sano Y, Kobayashi M, Hamamoto Y (2001). New diagnostic method based on color imaging using narrowband imaging (NBI) endoscopy system for gastrointestinal tract. *Gastrointestinal Endoscopy*.

[2] Piazza C, Dessouky O, Peretti G, Cocco D, De Benedetto L, Nicolai P (2008). Narrow-band imaging: a new tool for evaluation of head and neck squamous cell carcinomas. Review of the literature. *Acta Otorhinolaryngologica Italica *.

[3] Gono K, Cohen J (2007). An introduction to high-resolution endoscopy and narrowband imaging. *Advanced Digestive Endoscopy: Comprehensive Atlas of High Resolution Endoscopy and Narrowband Imaging*.

[4] Piazza C, Cocco D, De Benedetto L, Del Bon F, Nicolai P, Peretti G (2010). Narrow band imaging and high definition television in the assessment of laryngeal cancer: a prospective study on 279 patients. *European Archives of Oto-Rhino-Laryngology*.

[5] Watanabe A, Taniguchi M, Tsujie H, Hosokawa M, Fujita M, Sasaki S (2009). The value of narrow band imaging for early detection of laryngeal cancer. *European Archives of Oto-Rhino-Laryngology*.

[6] Inoue H, Kaga M, Sato Y, Sugaya S, Kudo S, Cohen J (2007). Magnifying endoscopic diagnosis of tissue atypia and cancer invasion depth in the area of pharyngo-esophageal squamous epithelium by NBI enhanced magnification image: IPCL pattern classification. *Advanced Digestive Endoscopy: Comprehensive Atlas of High Resolution Endoscopy and Narrowband Imaging*.

[7] Kumagai Y, Inoue H, Nagai K, Kawano T, Iwai T (2002). Magnifying endoscopy, stereoscopic microscopy, and the microvascular architecture of superficial esophageal carcinoma. *Endoscopy*.

[8] Piazza C, del Bon F, Peretti G, Nicolai P (2012). Narrow band imaging in endoscopic evaluation of the larynx. *Current Opinion in Otolaryngology & Head and Neck Surgery*.

[9] Lukeš P, Amornyotin S (2013). Narrow Band Imaging (NBI)—endoscopic method for detection of head and neck cancer. *Endoscopy*.

[10] Takano JH, Yakushiji T, Kamiyama I (2010). Detecting early oral cancer: narrowband imaging system observation of the oral mucosa microvasculature. *International Journal of Oral and Maxillofacial Surgery*.

[11] Ni X-G, He S, Xu Z-G (2011). Endoscopic diagnosis of laryngeal cancer and precancerous lesions by narrow band imaging. *Journal of Laryngology and Otology*.

[12] Hanahan D, Folkman J (1996). Patterns and emerging mechanisms of the angiogenic switch during tumorigenesis. *Cell*.

[13] Tjon Pian Gi RE, Halmos GB, van Hemel BM (2012). Narrow band imaging is a new technique in visualization of recurrent respiratory papillomatosis. *Laryngoscope*.

[14] Andrea M, Dias O, Santos A (1995). Contact endoscopy during microlaryngeal surgery: a new technique for endoscopic examination of the larynx. *Annals of Otology, Rhinology and Laryngology*.

[15] Rodríguez-Caso L, Reyes-Palomares A, Sánchez-Jiménez F, Quesada AR, Ángel Medina M (2012). What is known on angiogenesis-related rare diseases? A systematic review of literature. *Journal of Cellular and Molecular Medicine*.

[16] Bolontrade MF, Stern MC, Binder RL, Zenklusen JC, Gimenez-Conti IB, Conti CJ (1998). Angiogenesis is an early event in the development of chemically induced skin tumors. *Carcinogenesis*.

